# Supplementary material for: Bodiniosides S–Y, Seven New Triterpenoid Saponins from Elsholtzia bodinieri and Their Anti-Influenza Activities
Source: Molecules. 2021 Oct 29;26(21):6535. doi: 10.3390/molecules26216535 (PMC8587758; doi:10.3390/molecules26216535)
Supplement: Supplementary file 1 [file molecules-26-06535-s001.zip › molecules-1412501-supplementary.pdf]

SUPPLEMENTARY MATERIAL

Bodiniosides S – Y, Seven New Triterpenoid Saponins from *Elsholtzia bodinieri* and Their Anti-Influenza Activities

Lin-Yao Yang[1†], Jiang-Chao Du[1†], Rong-Tao Li[1], Fei Yu[2], and Jin-Dong Zhong[1*]

[1]Faculty of Life Science and Technology, Kunming University of Science and Technology, Kunming, 650500, China; E-Mails: linyaoyang0808@163.com (L.-Y. Y); dujiangchao2021@163.com (J.-C. D); rongtaolikm@163.com (R.-T. L);

[2]Medical School, Kunming University of Science and Technology, Kunming, Yunnan, China; E-Mails: feiyuz8@163.com(F. Y)

* Correspondence: jindongzhongkm@163.com; Tel：+86-871-65916009

[†]These authors contributed equally to this work.

Abstract

Investigation of the n-BuOH extract of the aerial parts of *Elsholtzia bodinieri* led to the isolation of seven new triterpenoid saponins, Bodiniosides S - Y (**1** – **7**, resp.). Their strictures were elucidated on the basis of spectroscopic techniques, including HSQC, HSBC, and HSQC-TOCSY experiments, together with acid hydrolysis and GC analysis. Anti-influenza activities of compounds **1** - **7** were evaluated against A/WSN/33/2009 (H1N1) virus in MDCK cells. As the results, compounds **2** and **5** exhibited potent anti-influenza activities against A/WSN/33/2009 (H1N1), with inhibition rate of 35.33% and 24.08%, respectively.

Keywords: *Elsholtzia bodinieri*; triterpenoid saponins; anti-influenza virus activities; Bodiniosides S – Y;

CONTACT Jin-Dong Zhong, email: jindongzhongkm@163.com

List of Contents

Figure S1 Key HMBC correlation of compound **1**
Figure S2 ^{1}H NMR spectrum of compound **1**(600 MHz, pyridine-d_5)
Figure S3 ^{13}C NMR spectrum of compound **1**(150 MHz, pyridine-d_5)
Figure S4 HSQC spectrum of compound **1**
Figure S5 HMBC spectrum of compound **1**
Figure S6 HRESIMS spectrum of compound **1**
Figure S7 Key HMBC correlation of compound **2**
Figure S8 ^{1}H NMR spectrum of compound **2**(600 MHz, pyridine-d_5)
Figure S9 ^{13}C NMR spectrum of compound **2**(150 MHz, pyridine-d_5)
Figure S10 HSQC spectrum of compound **2**
Figure S11 HMBC spectrum of compound **2**
Figure S12 HRESIMS spectrum of compound **2**
Figure S13 Key HMBC correlation of compound **3**
Figure S14 ^{1}H NMR spectrum of compound **3** (600 MHz, pyridine-d_5)
Figure S15 ^{13}C NMR spectrum of compound **3**(150 MHz, pyridine-d_5)
Figure S16 HSQC spectrum of compound **3**
Figure S17 HMBC spectrum of compound **3**
Figure S18 HRESIMS spectrum of compound **3**
Figure S19 Key HMBC correlation of compound **4**
Figure S20 ^{1}H NMR spectrum of compound **4** (600 MHz, pyridine-d_5)
Figure S21 ^{13}C NMR spectrum of compound **4**(150 MHz, pyridine-d_5)
Figure S22 HSQC spectrum of compound **4**
Figure S23 HMBC spectrum of compound **4**
Figure S24 HRESIMS spectrum of compound **4**
Figure S25 Key HMBC correlation of compound **5**
Figure S26 ^{1}H NMR spectrum of compound **5** (600 MHz, pyridine-d_5)
Figure S27 ^{13}C NMR spectrum of compound **5**(150 MHz, pyridine-d_5)
Figure S28 HSQC spectrum of compound **5**
Figure S29 HMBC spectrum of compound **5**
Figure S30 HRESIMS spectrum of compound **5**
Figure S31 Key HMBC correlation of compound **6**
Figure S32 ^{1}H NMR spectrum of compound **6** (600 MHz, pyridine-d_5)
Figure S33 ^{13}C NMR spectrum of compound **6**(150 MHz, pyridine-d_5)
Figure S34 HSQC spectrum of compound **6**
Figure S35 HMBC spectrum of compound **6**
Figure S36 HRESIMS spectrum of compound **6**
Figure S37 Key HMBC correlation of compound **7**
Figure S38 ^{1}H NMR spectrum of compound **7** (600 MHz, pyridine-d_5)
Figure S39 ^{13}C NMR spectrum of compound **7**(150 MHz, pyridine-d_5)
Figure S40 HSQC spectrum of compound **7**
Figure S41 HMBC spectrum of compound **7**
Figure S42 HRESIMS spectrum of compound **7**

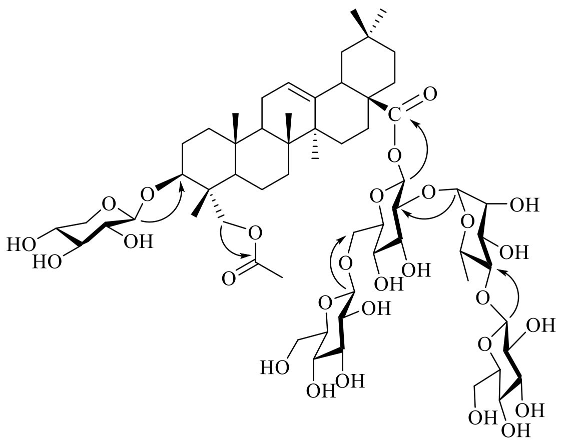

Figure S1 Key HMBC correlation of compound **1**

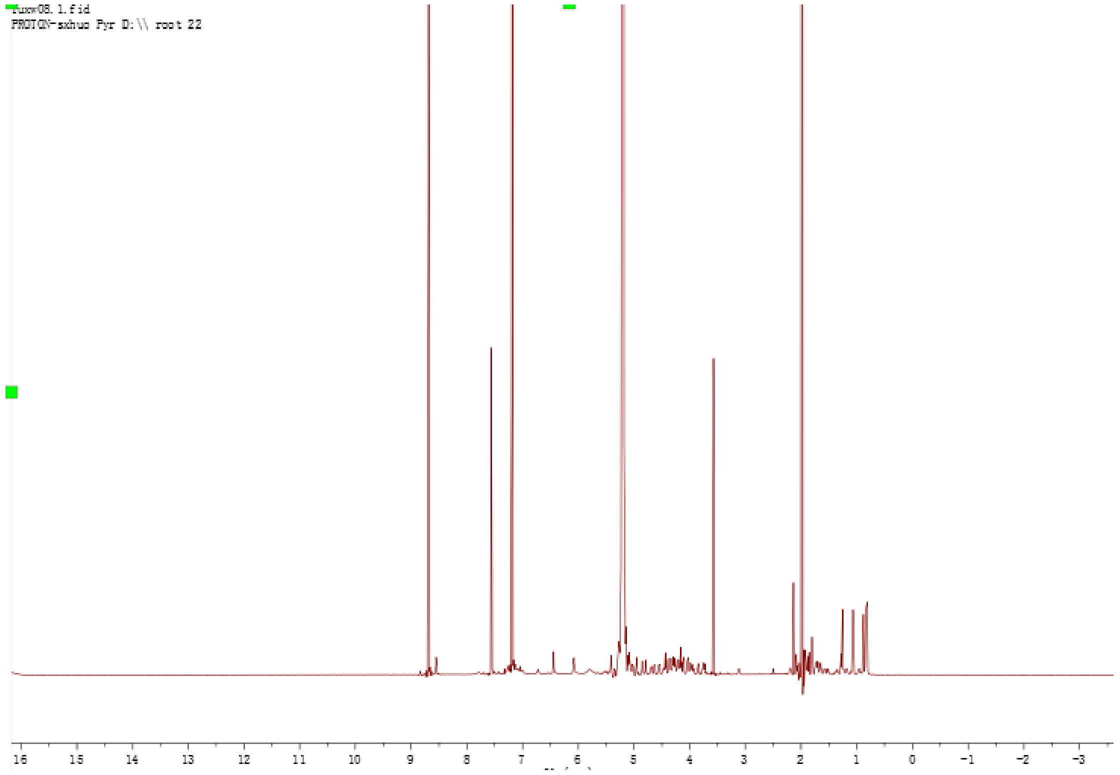

Figure S2 ^{1}H NMR spectrum of compound **1**(600 MHz, pyridine-d_5)

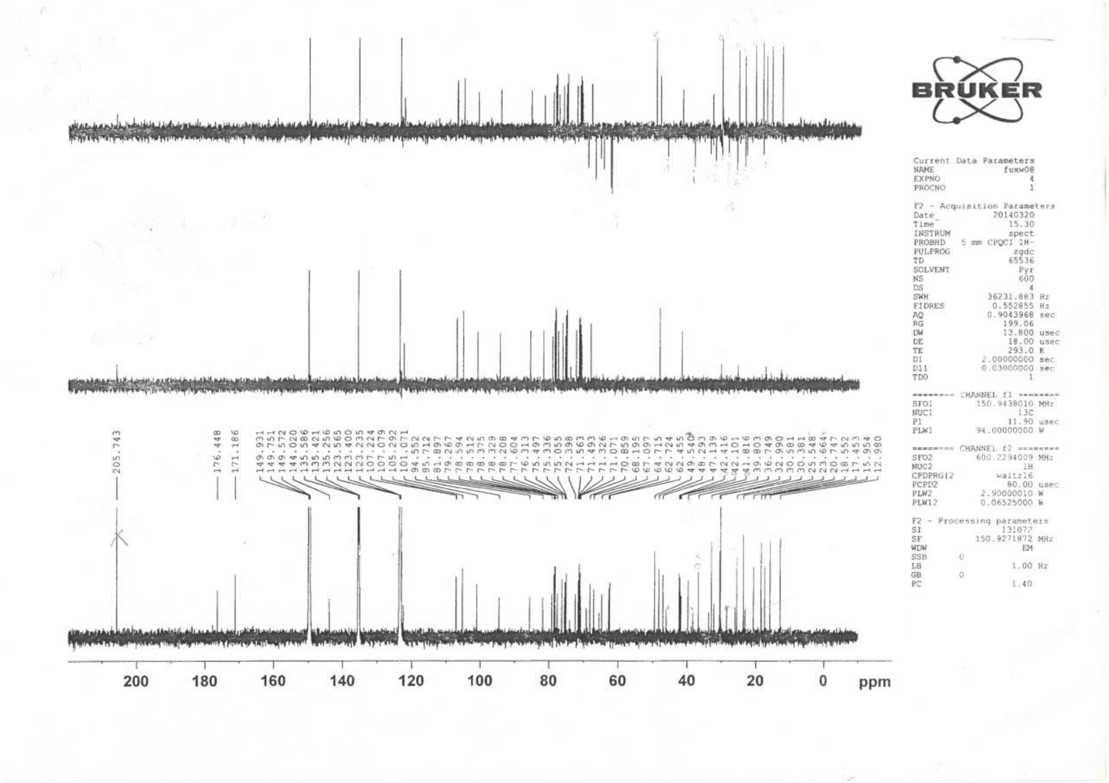

Figure S3 ^{13}C NMR spectrum of compound **1**(150 MHz, pyridine-d_5)

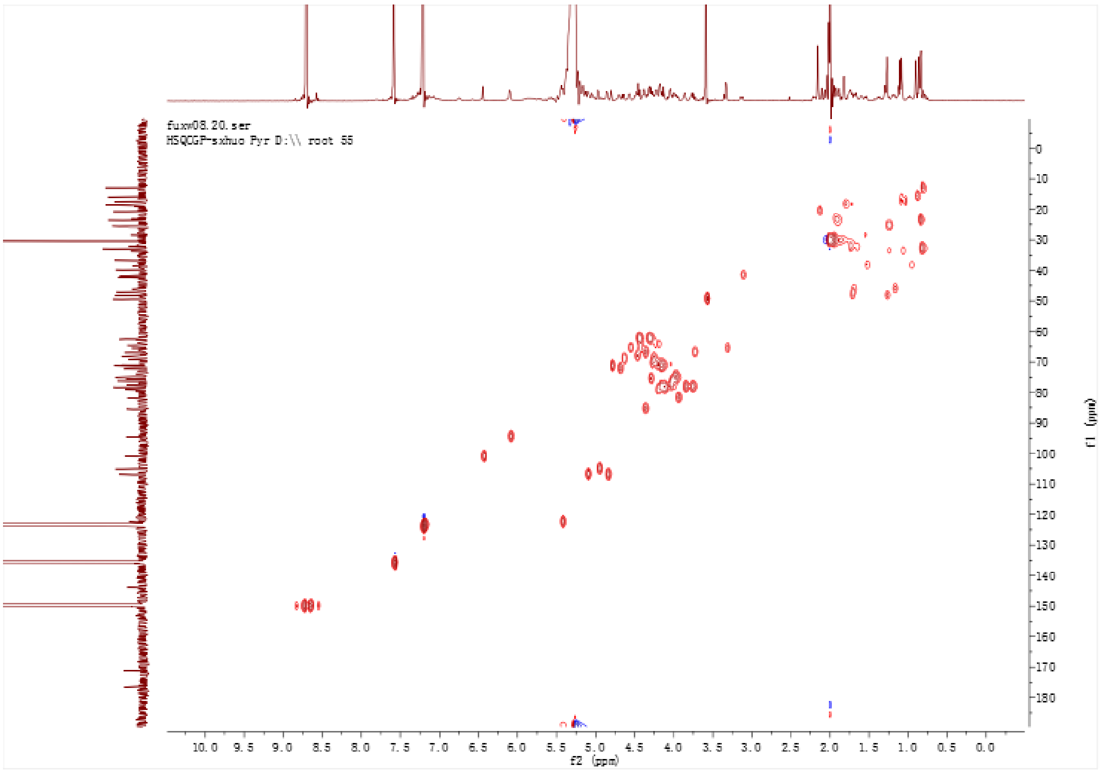

Figure S4 HSQC spectrum of compound **1**

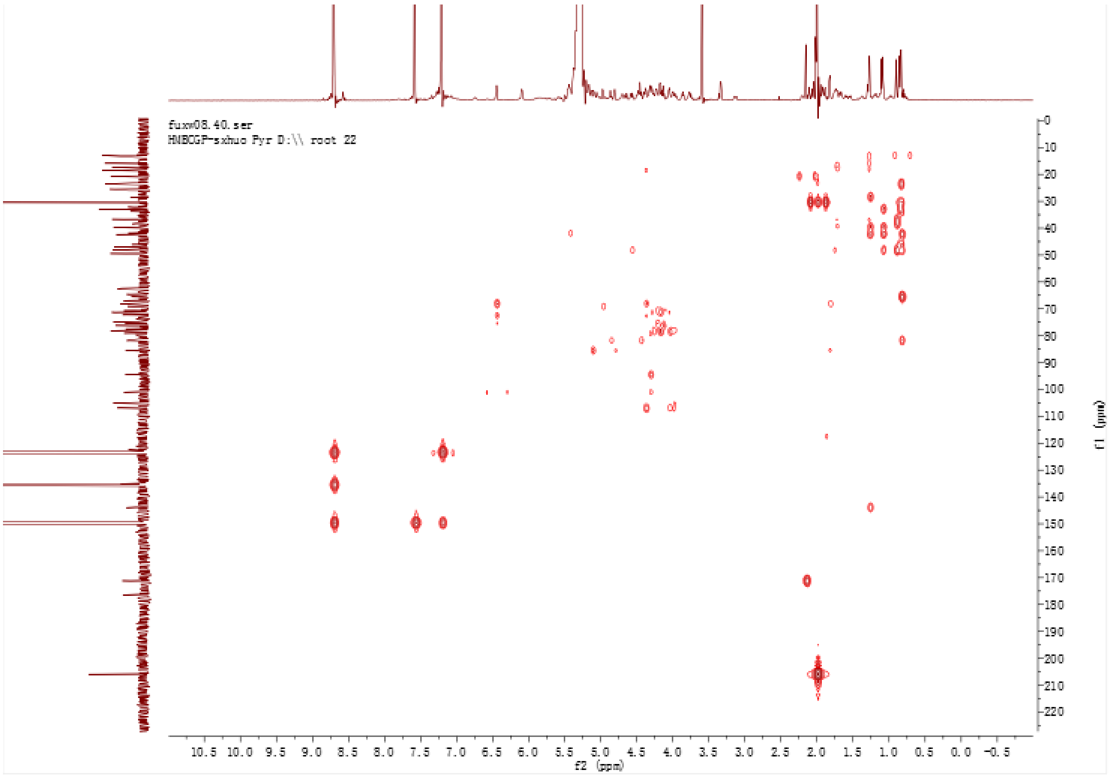

Figure S5 HMBC spectrum of compound **1**

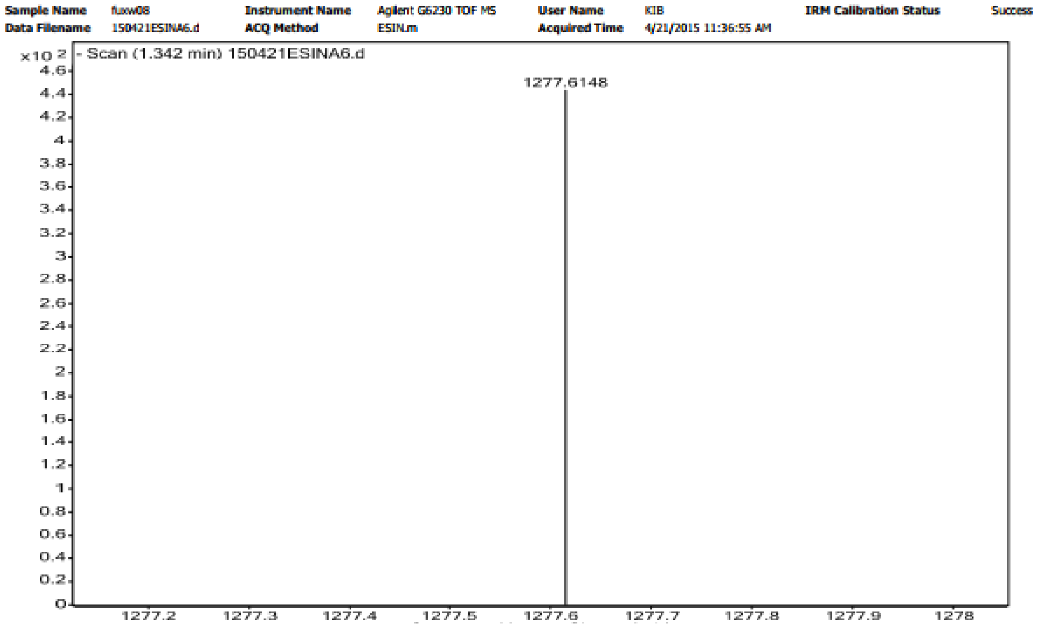

Figure S6 HRESIMS spectrum of compound **1**

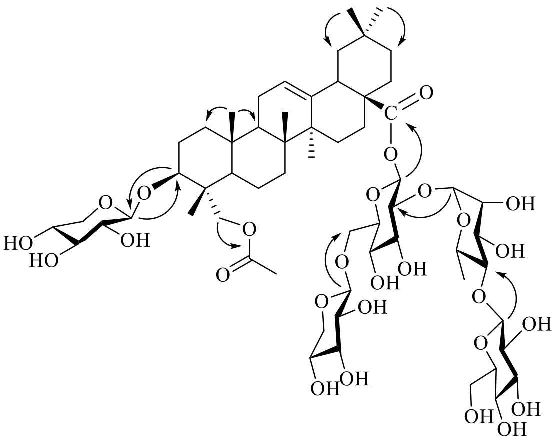

Figure S7 Key HMBC correlation of compound **2**

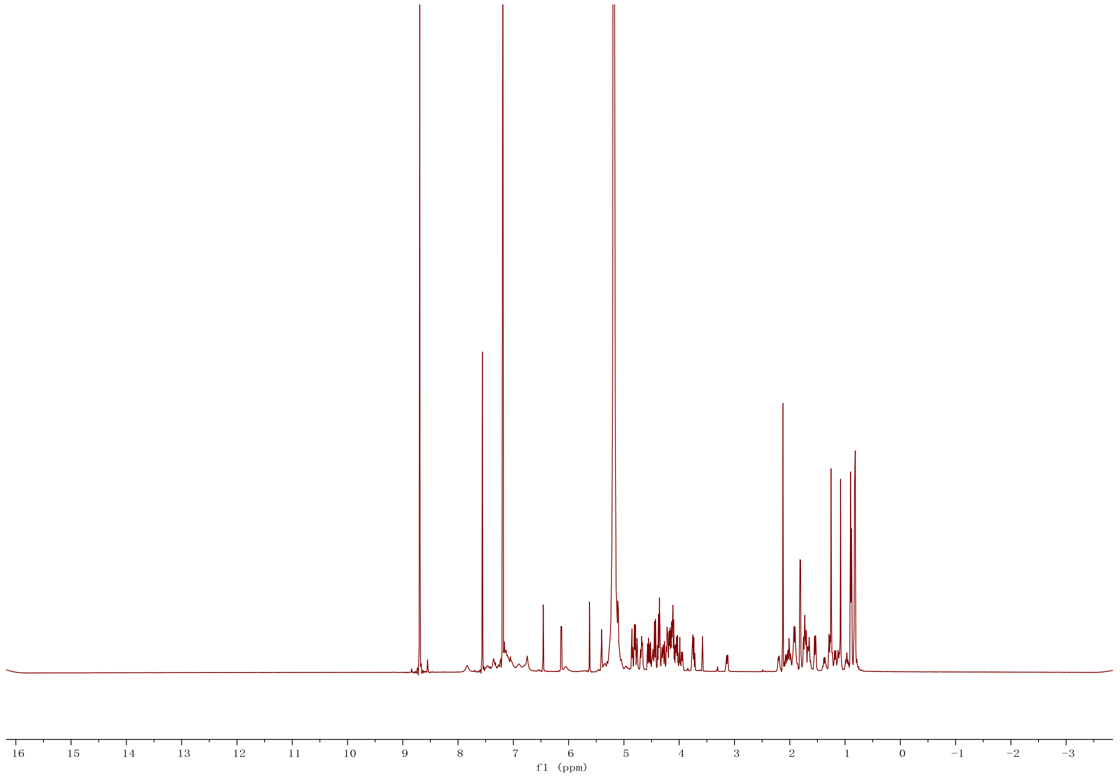

Figure S8 ^{1}H NMR spectrum of compound **2**(600 MHz, pyridine-d_5)

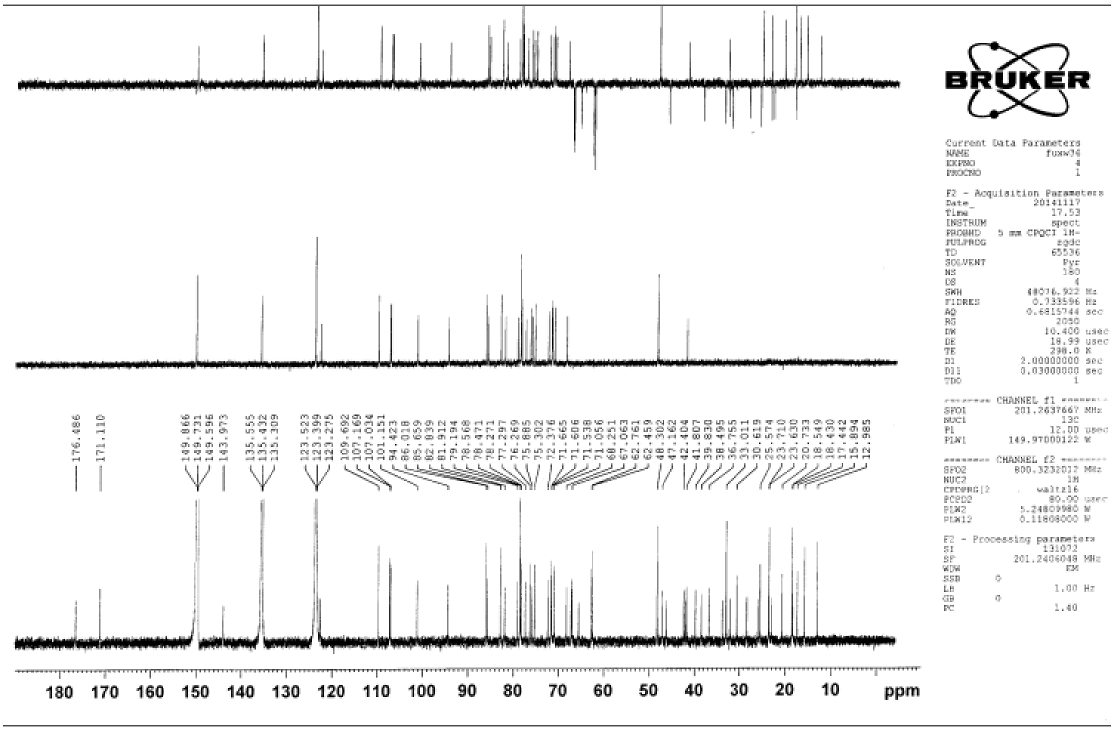

Figure S9 ^{13}C NMR spectrum of compound **2**(150 MHz, pyridine-d_5)

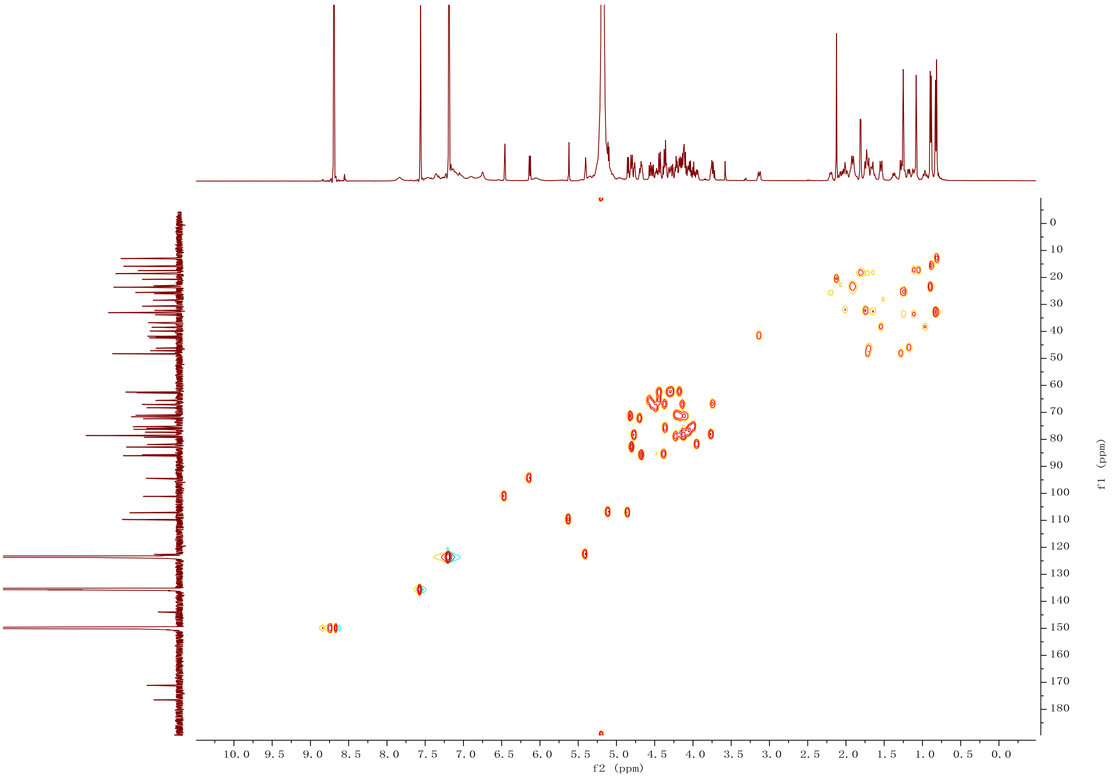

Figure S10 HSQC spectrum of compound **2**

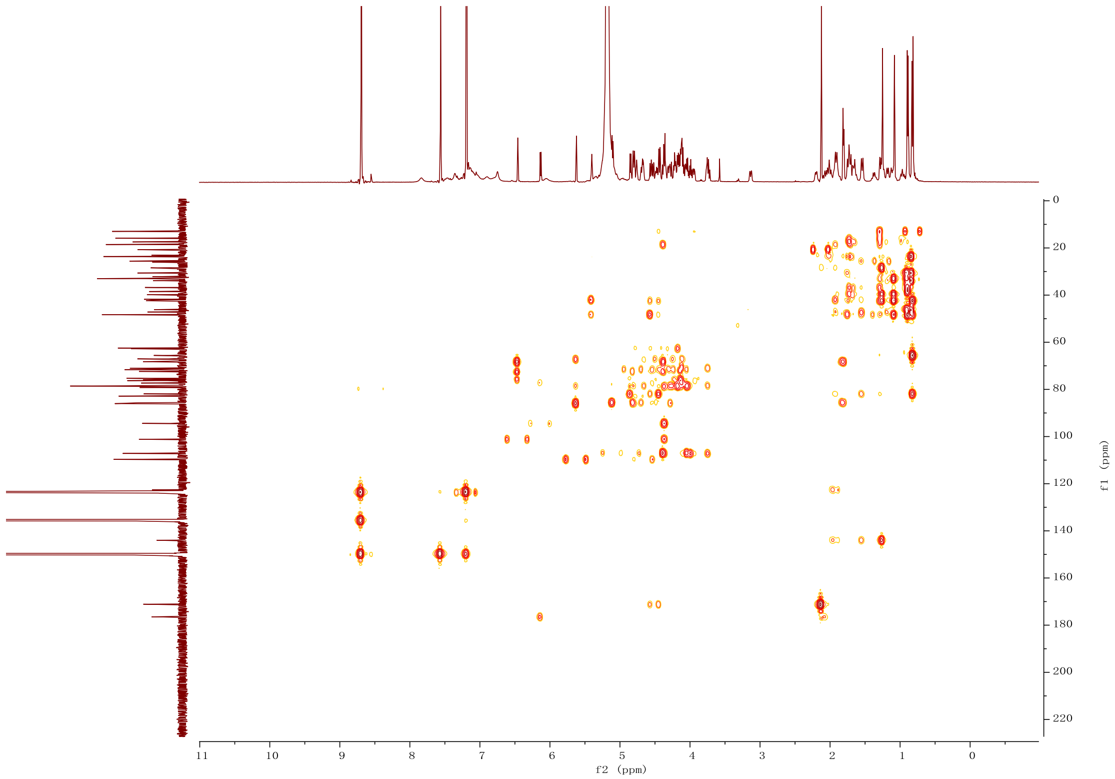

Figure S11 HMBC spectrum of compound **2**

Qualitative Analysis Report

**Data Filename**	fuxw36.d	**Sample Name**	fuxw36
**Sample Type**	Sample	**Position**	P1-A7
**Instrument Name**	Instrument 1	**User Name**	
**Acq Method**	SIBU-ESI-.m	**Acquired Time**	9/22/2015 3:56:44 PM
**IRM Calibration Status**	Success	**DA Method**	ESI+.m
**Comment**			

Sample Group **Info.**
Acquisition SW 6200 series TOF/6500 series
Version Q-TOF B.05.01 (B5125.2)

User Spectra

Fragmentor Voltage	Collision Energy	Ionization Mode
135	0	ESI

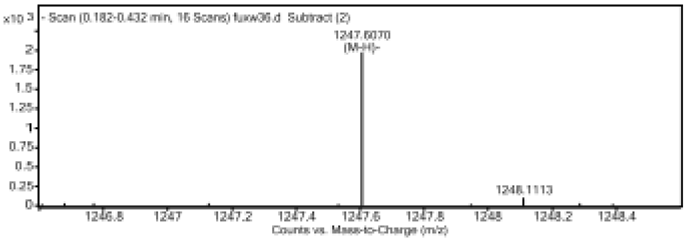

Peak List

m/z	z	Abund	Formula	Ion
623.2991	2	1121.34		
646.3021	2	1139.5		
646.805	2	870.68		
1247.607	1	1958.22	C60 H96 O27	(M-H)-
1248.608	1	1383.56	C60 H96 O27	(M-H)-
1293.6099	1	913.5		
1361.5953	1	1281.46		
1362.5957	1	773.62		

Formula Calculator Element Limits

Element	Min	Max
C	3	60
H	0	120
O	0	60

Formula Calculator Results

Formula	CalculatedMass	CalculatedMz	Mz	Diff. (mDa)	Diff. (ppm)	DBE
C60 H96 O27	1248.6139	1247.6066	1247.6070	0.8	0.6	13.0000

--- End Of Report ---

Figure S12 HRESIMS spectrum of compound **2**

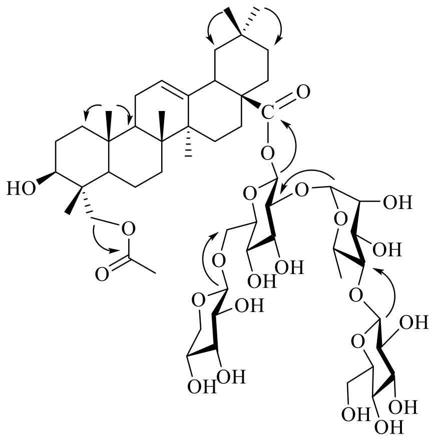

Figure S13 Key HMBC correlation of compound **3**

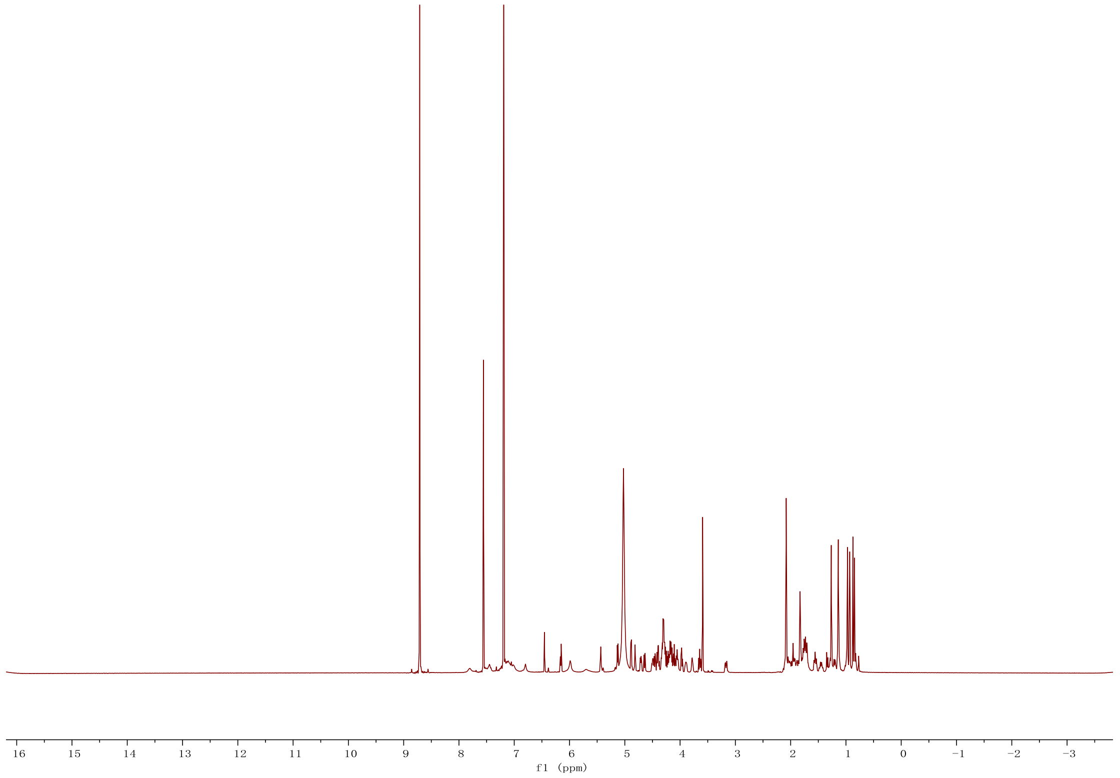

Figure S14 ^{1}H NMR spectrum of compound **3** (600 MHz, pyridine-d_5)

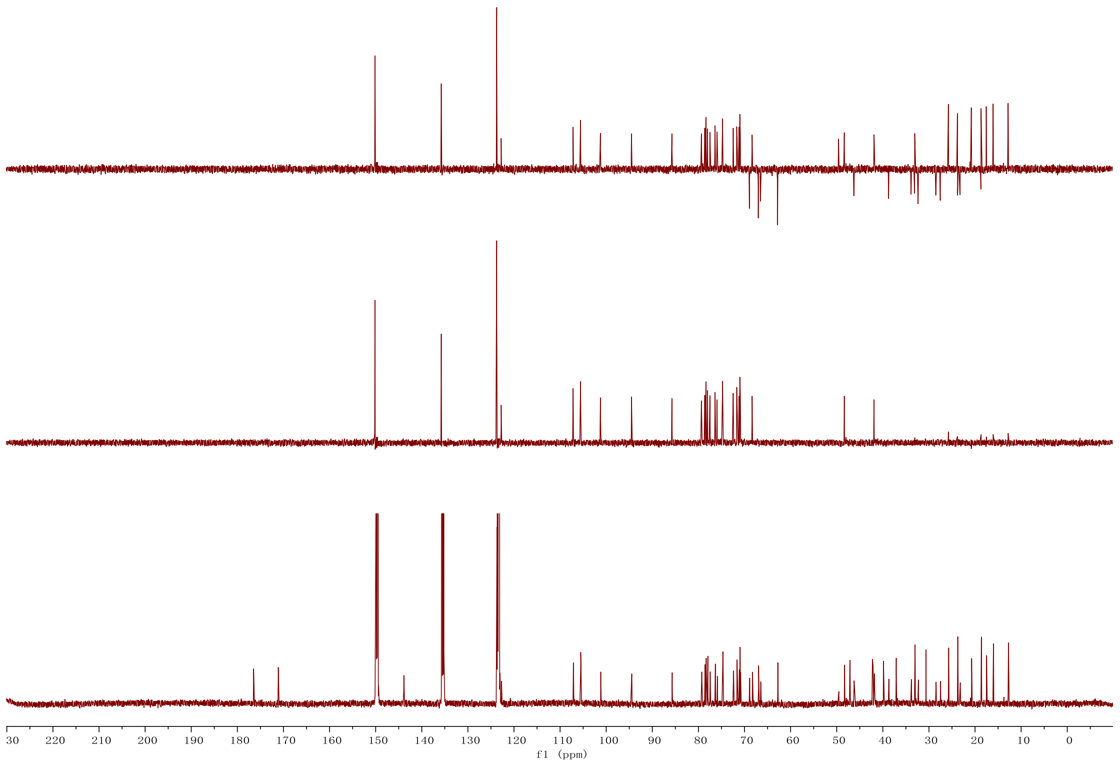

Figure S15 ^{13}C NMR spectrum of compound **3**(150 MHz, pyridine-d_5)

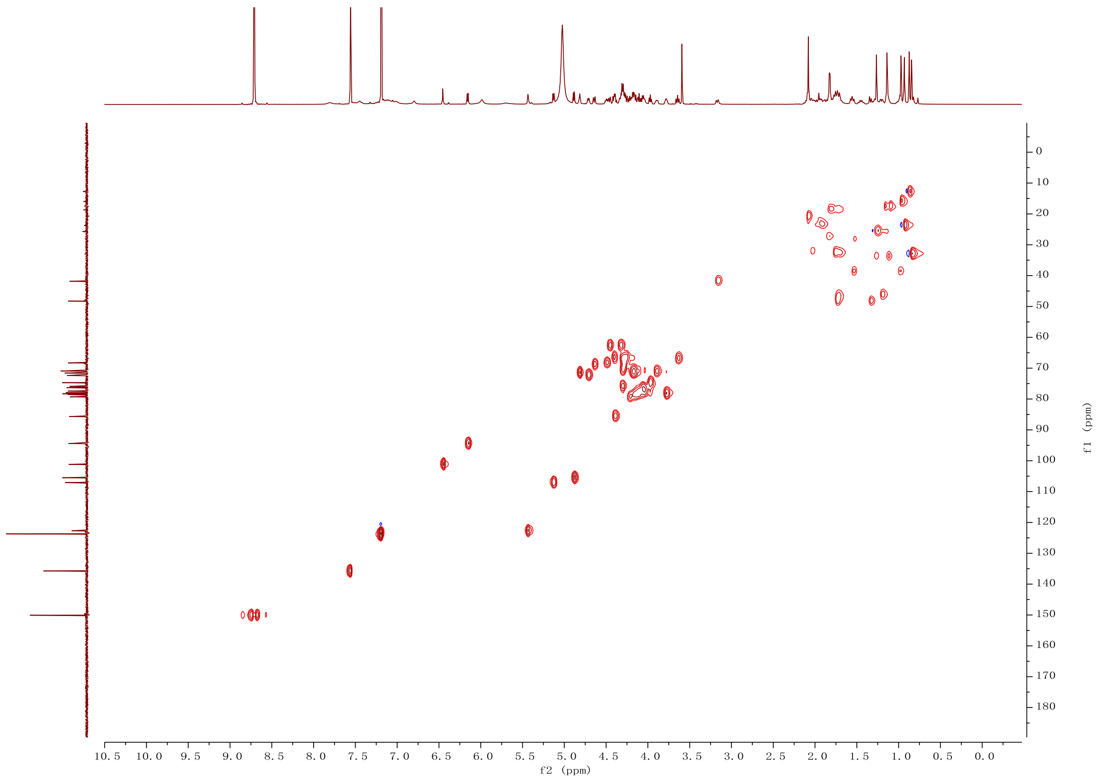

Figure S16 HSQC spectrum of compound **3**

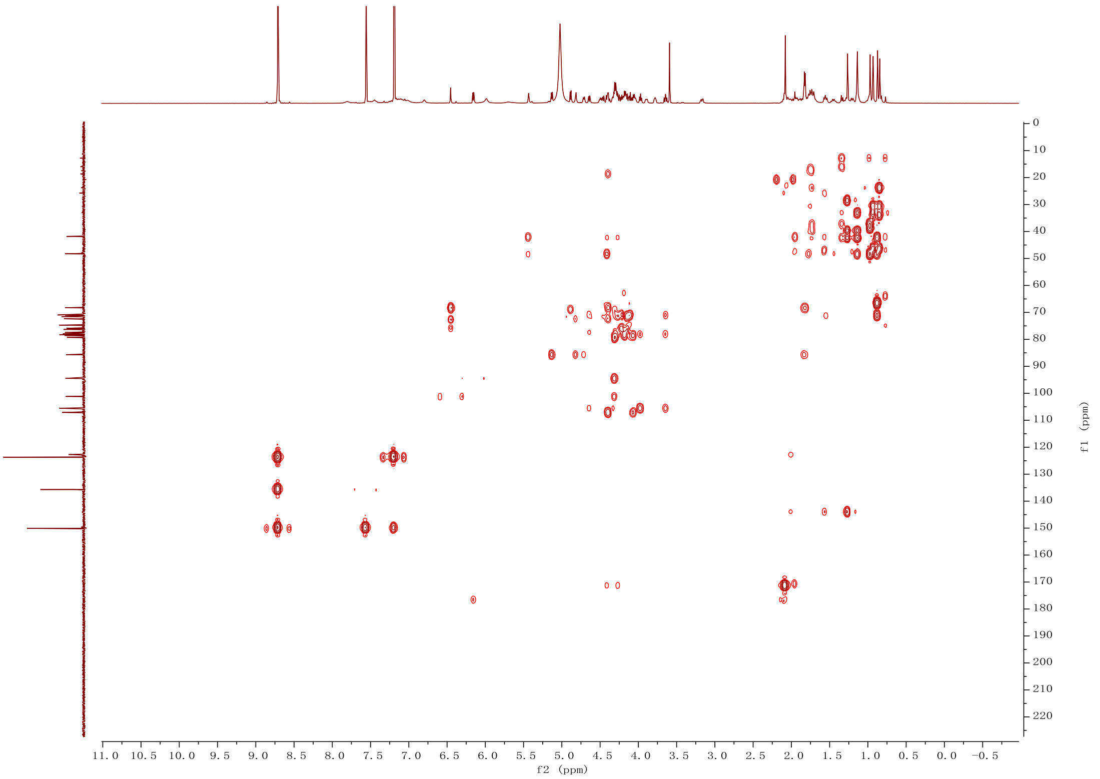

Figure S17 HMBC spectrum of compound **3**

Qualitative Analysis Report

**Data Filename**	fuxw43+.d	**Sample Name**	fuxw43
**Sample Type**	Sample	**Position**	P1-D6
**Instrument Name**	Instrument 1	**User Name**	
**Acq Method**	SIBU.m	**Acquired Time**	11/5/2015 10:23:27 AM
**IRM Calibration Status**	Success	**DA Method**	ESI+.m
**Comment**			

**Sample Group**		**Info.**
**Acquisition SW**	6200 series TOF/6500 series	
**Version**	Q-TOF B.05.01 (B5125.2)	

User Spectra

Fragmentor Voltage	Collision Energy	Ionization Mode
135	0	ESI

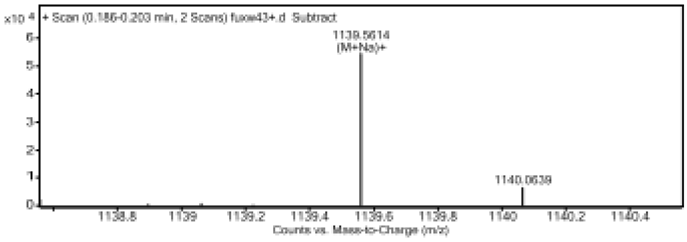

Peak List

m/z	z	Abund	Formula	Ion
570.2806	2	11468.18		
578.2691	2	33672.49		
578.7708	2	21226.05		
1139.5614	1	54369.45	C55 H88 O23	(M+Na)+
1140.565	1	32070.97	C55 H88 O23	(M+Na)+
1141.5679	1	11161.86	C55 H88 O23	(M+Na)+
1155.5347	1	15864.92		
1156.5377	1	9860.31		

Formula Calculator Element Limits

Element	Min	Max
C	3	60
H	0	120
O	0	30

Formula Calculator Results

Formula	CalculatedMass	CalculatedMz	Mz	Diff. (mDa)	Diff. (ppm)	DBE
C55 H88 O23	1116.5716	1139.5609	1139.5614	-0.5	-0.4	12.0000

Figure S18 HRESIMS spectrum of compound **3**

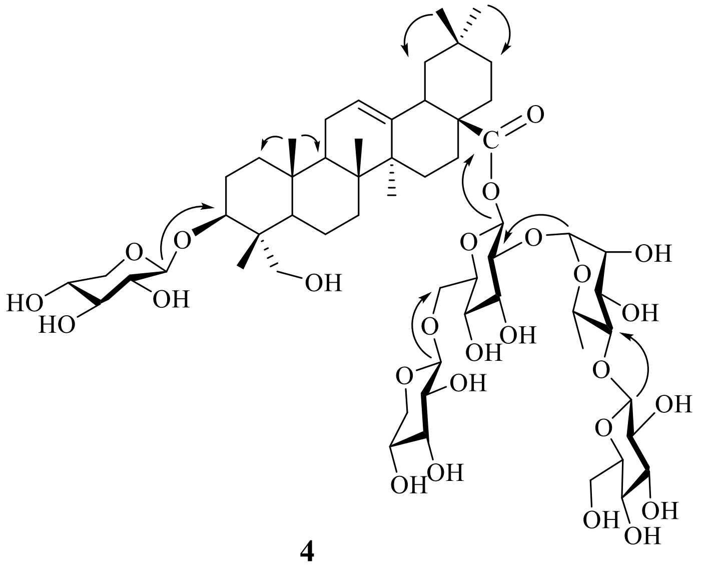

4

Figure S19 Key HMBC correlation of compound **4**

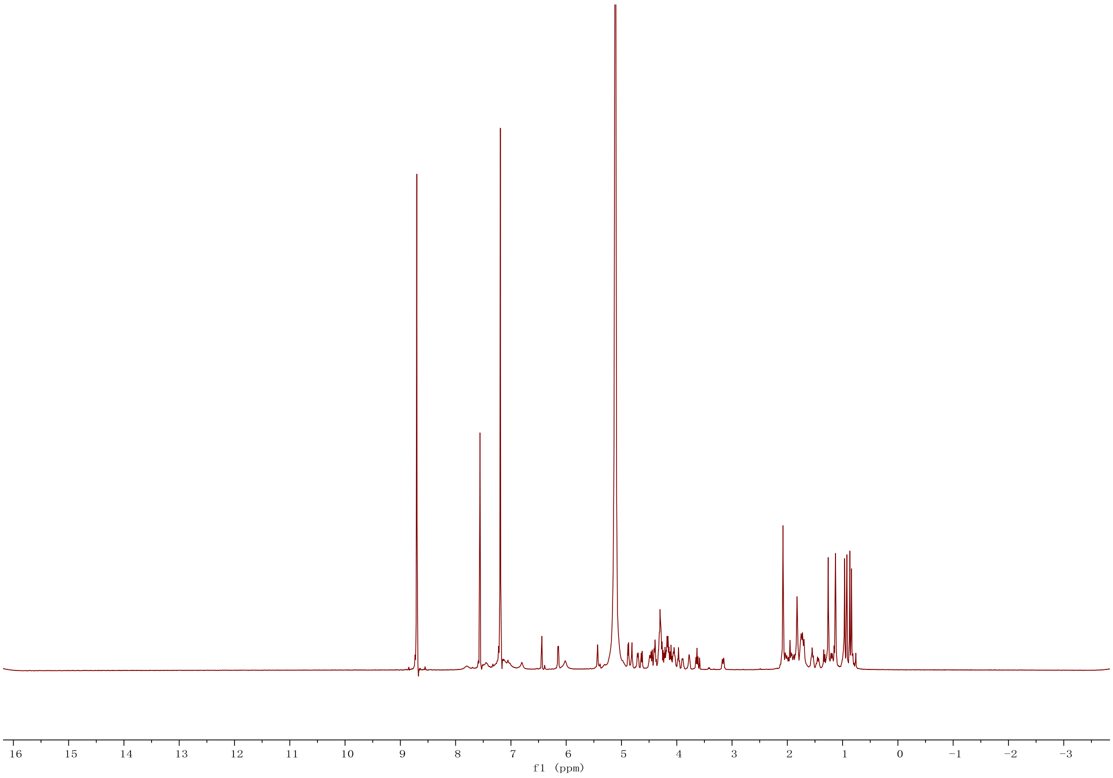

Figure S20 ^{1}H NMR spectrum of compound **4** (600 MHz, pyridine-d_5)

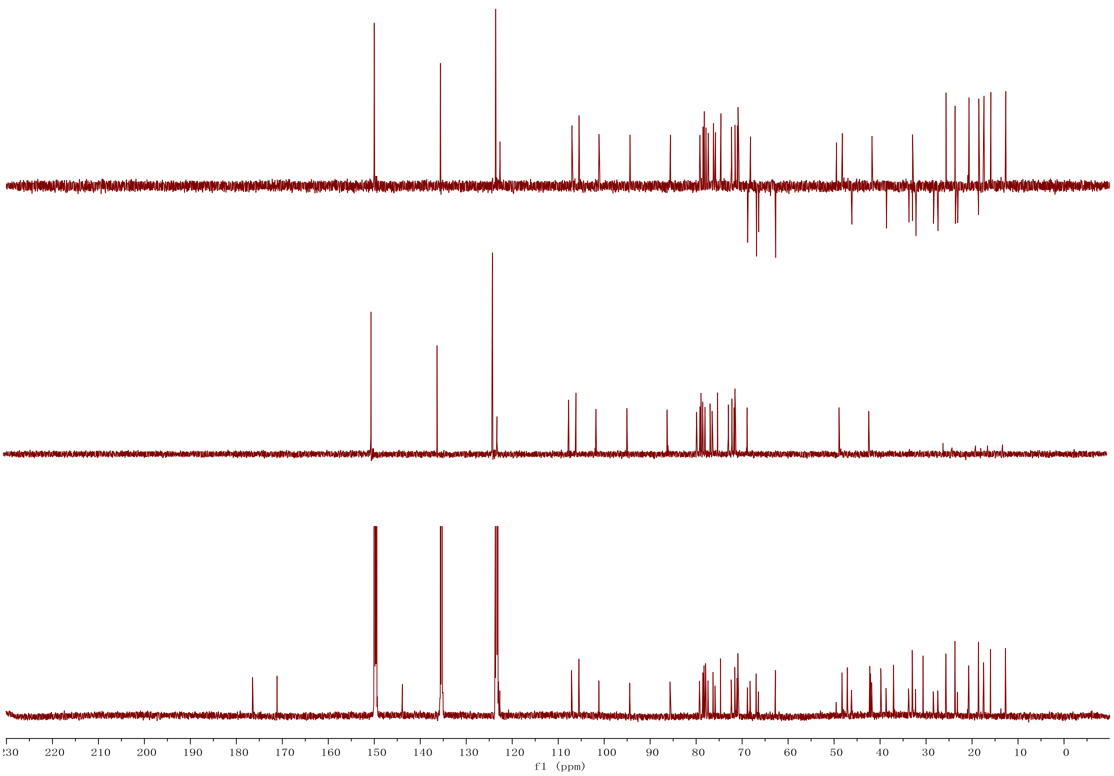

Figure S21 ^{13}C NMR spectrum of compound **4**(150 MHz, pyridine-d_5)

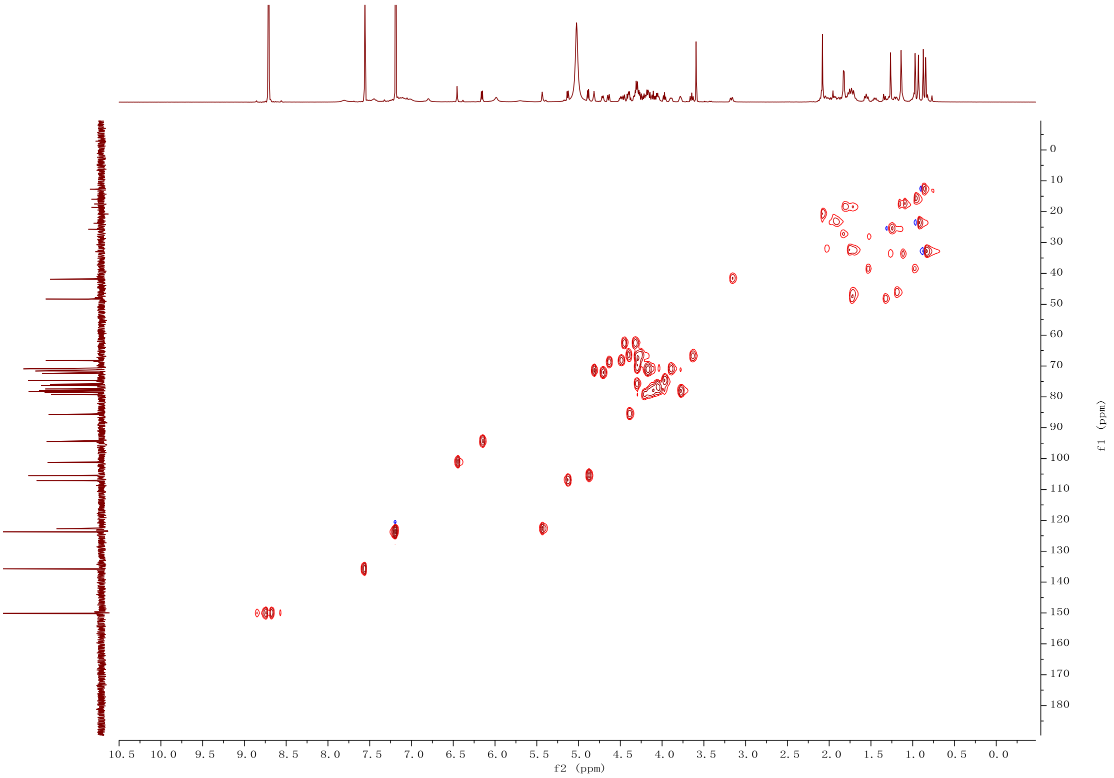

Figure S22 HSQC spectrum of compound **4**

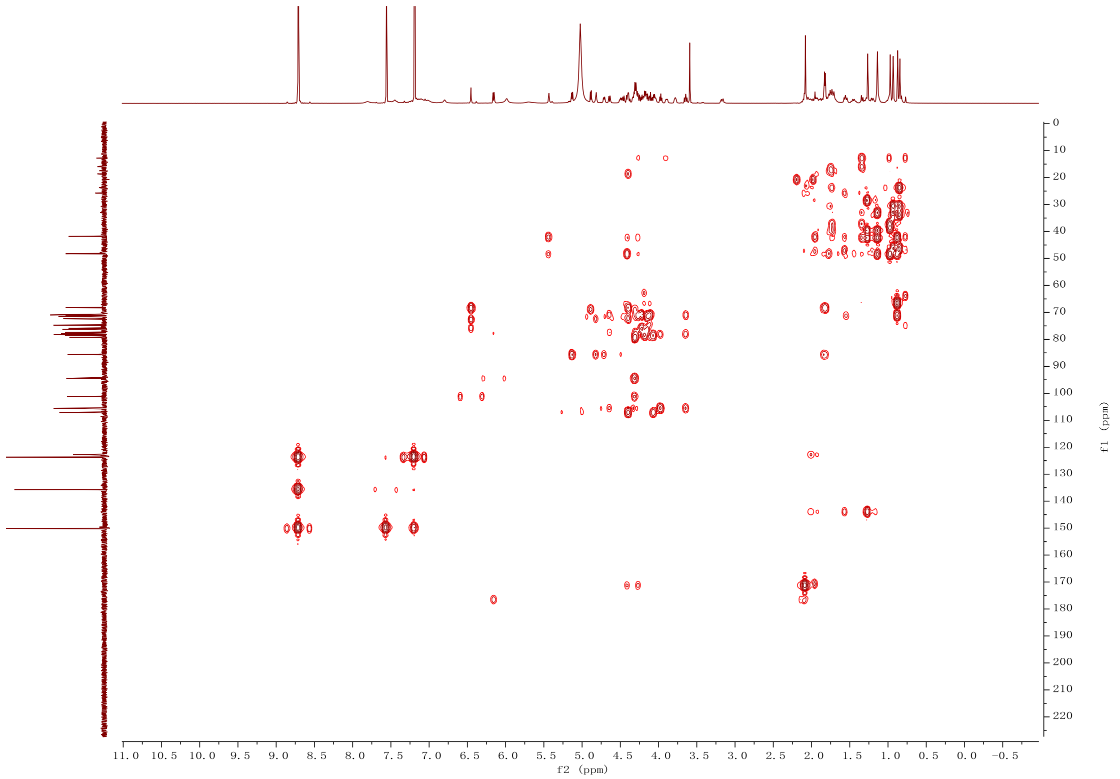

Figure S23 HMBC spectrum of compound **4**

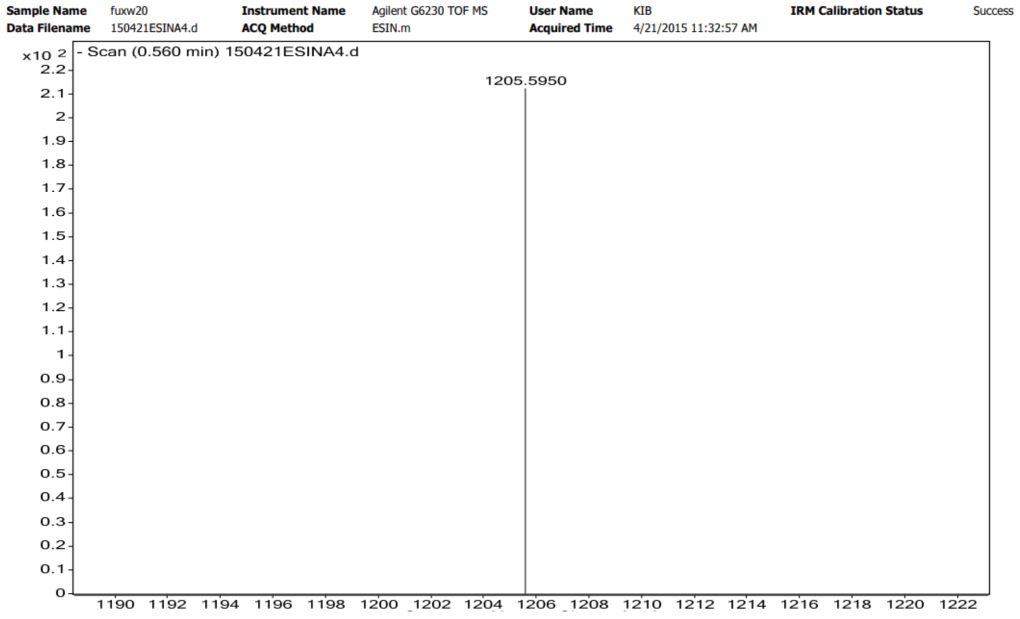

Figure S24 HRESIMS spectrum of compound **4**

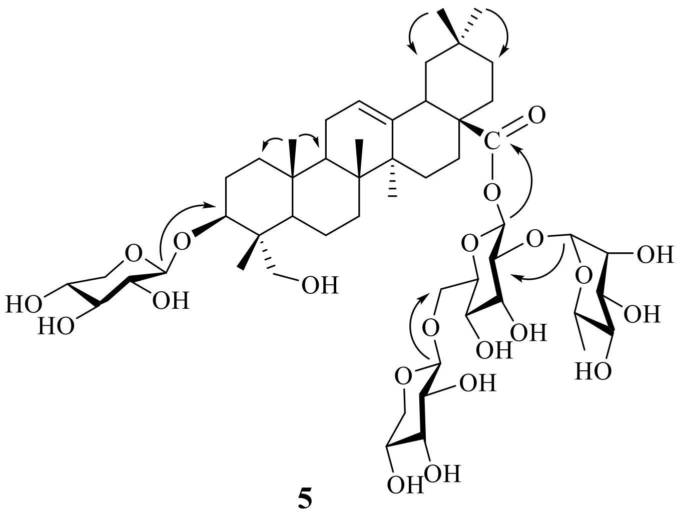

5

Figure S25 Key HMBC correlation of compound **5**

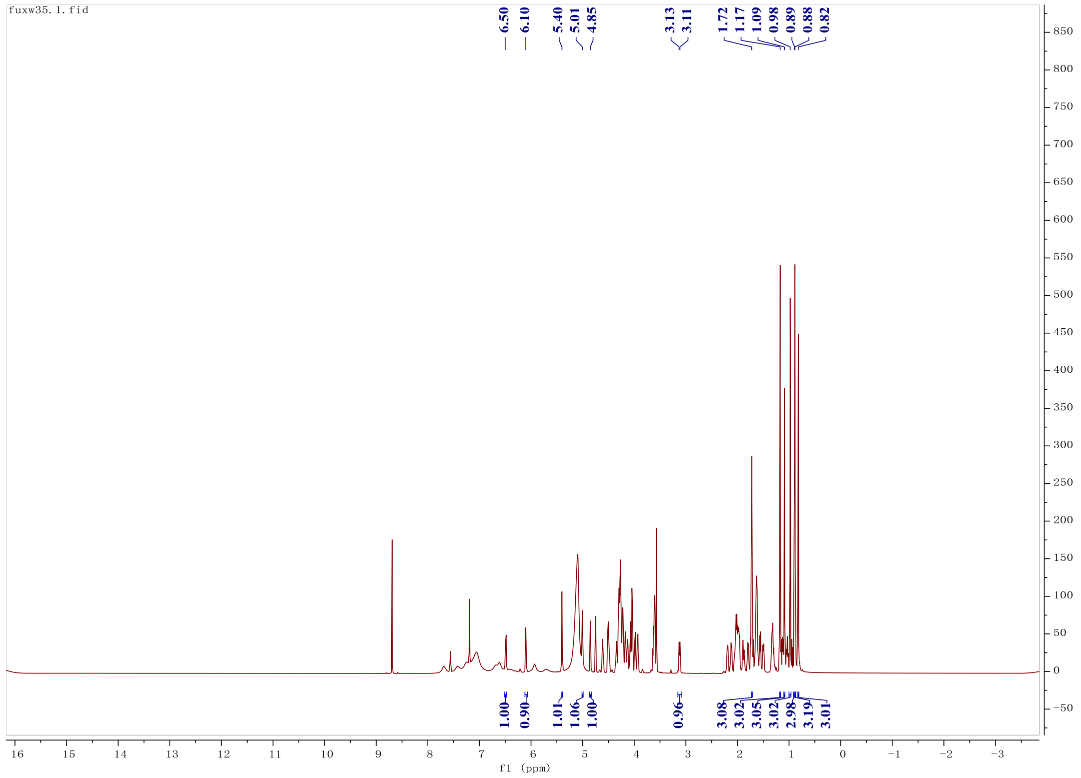

Figure S26 ^{1}H NMR spectrum of compound **5** (600 MHz, pyridine-d_5)

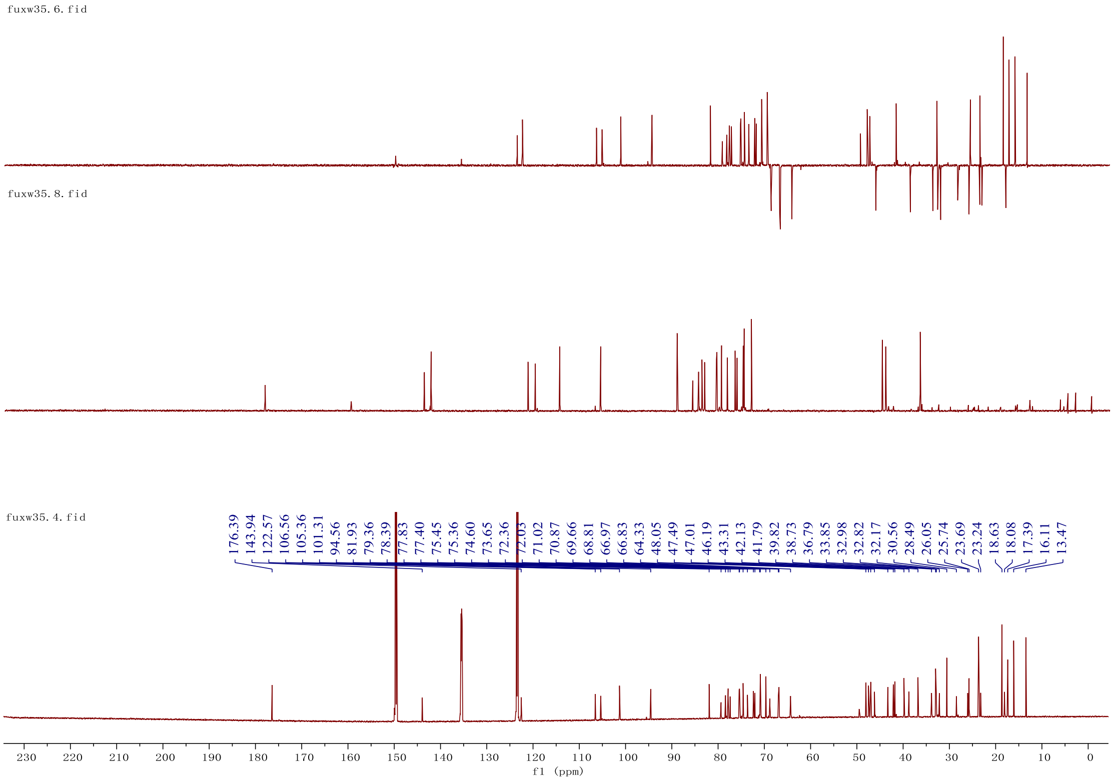

Figure S27 ^{13}C NMR spectrum of compound **5**(150 MHz, pyridine-d_5)

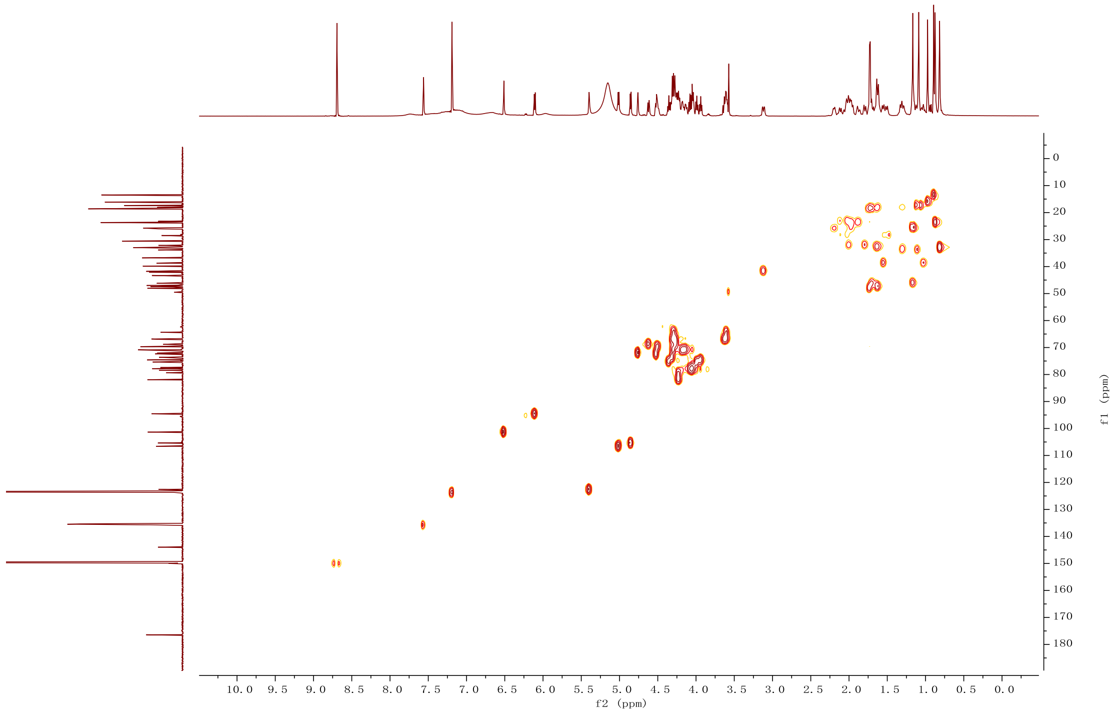

Figure S28 HSQC spectrum of compound **5**

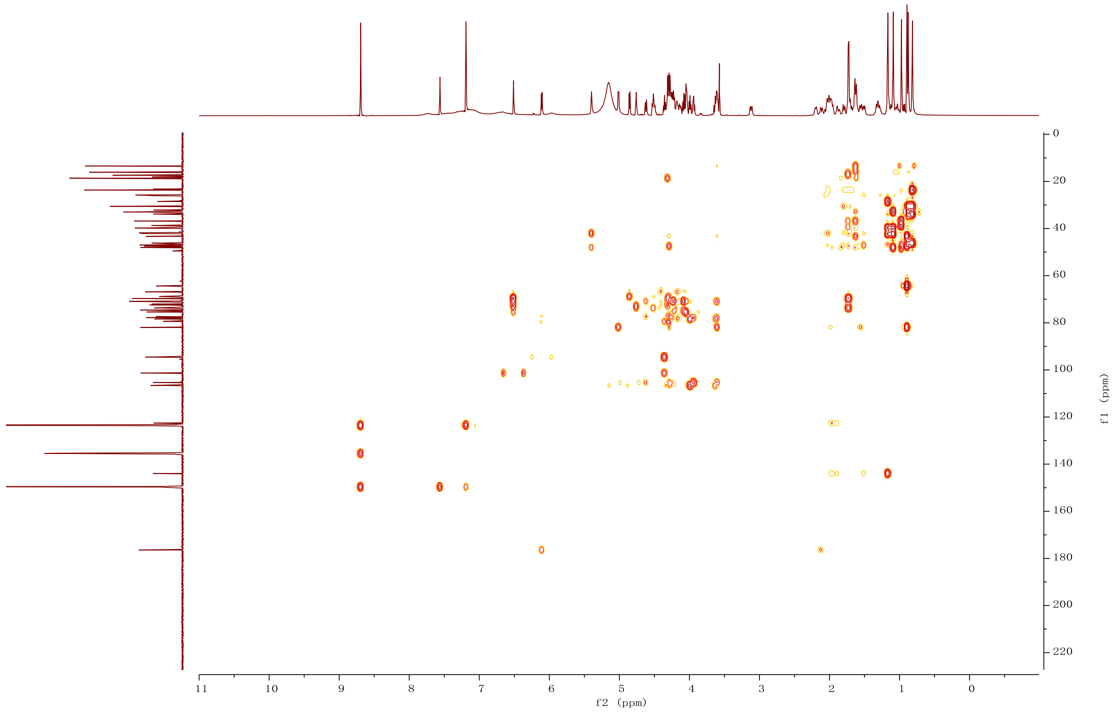

Figure S29 HMBC spectrum of compound **5**

Qualitative Analysis Report

**Data Filename**	fuxw35.d	**Sample Name**	fuxw35
**Sample Type**	Sample	**Position**	P1-A6
**Instrument Name**	Instrument 1	**User Name**	
**Acq Method**	SIBU-ESI-i.m	**Acquired Time**	9/22/2015 3:55:13 PM
**IRM Calibration Status**	Success	**DA Method**	ESI+.m
**Comment**			

Sample Group **Info.**
Acquisition SW 6200 series TOF/6500 series
Version Q-TOF B.05.01 (B5125.2)

User Spectra

Fragmentor Voltage	Collision Energy	Ionization Mode
135	0	ESI

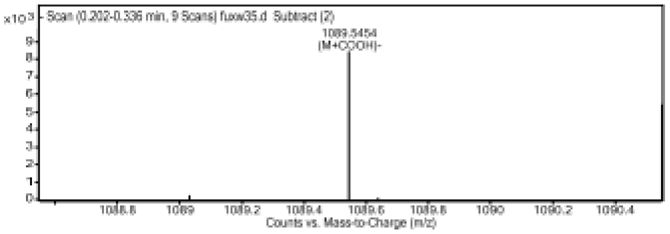

Peak List

m/z	z	Abund	Formula	Ion
362.9406	1	5479.9		
1043.5417	2	15203.24		
1044.546	2	8454.5		
1089.5454	1	8421.02	C52 H84 O21	(M+COOH)-
1090.5505	1	5362.31	C52 H84 O21	(M+COOH)-
1106.5393	1	6732.35		
1157.5343	1	13300.13		
1158.5396	1	7422.3		

Formula Calculator Element Limits

Element	Min	Max
C	3	60
H	0	120
O	0	60

Formula Calculator Results

Formula	CalculatedMass	CalculatedMz	Mz	Diff. (mDa)	Diff. (ppm)	DBE
C52 H84 O21	1044.5505	1089.5487	1089.5454	2.1	2.0	11.0000

Figure S30 HRESIMS spectrum of compound **5**

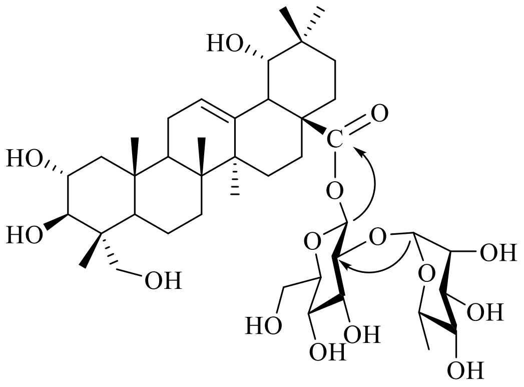

Figure S31 Key HMBC correlation of compound **6**

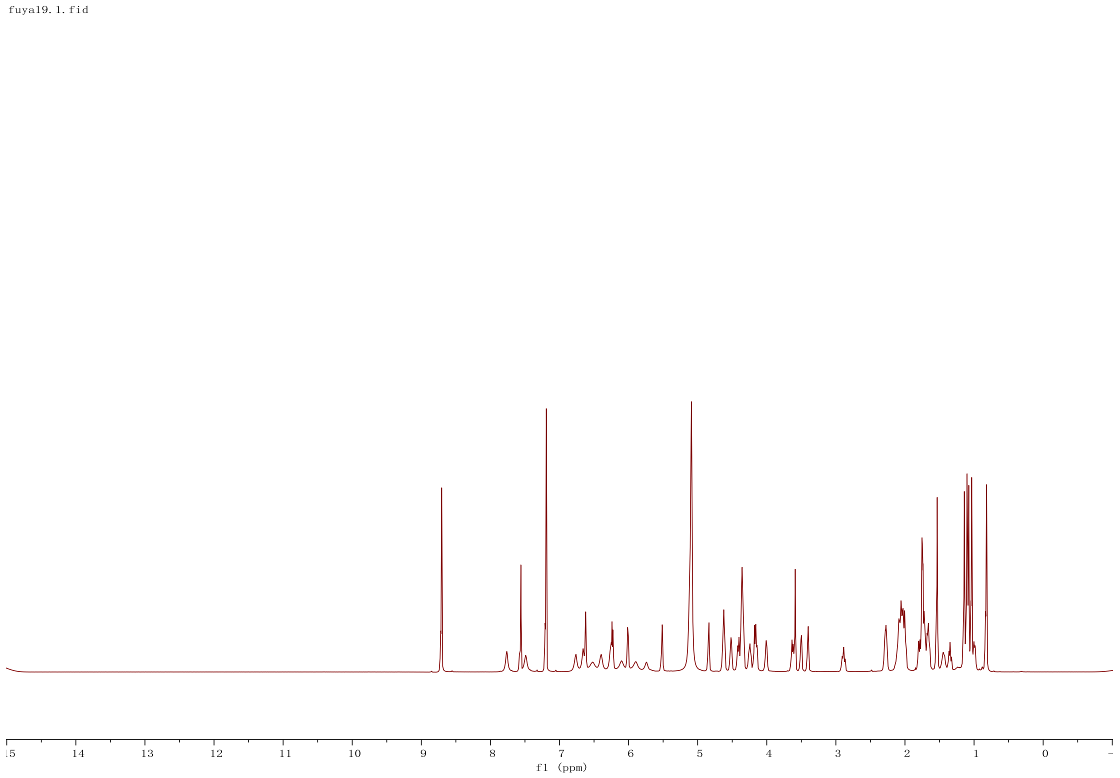

Figure S32 ^{1}H NMR spectrum of compound **6** (600 MHz, pyridine-d_5)

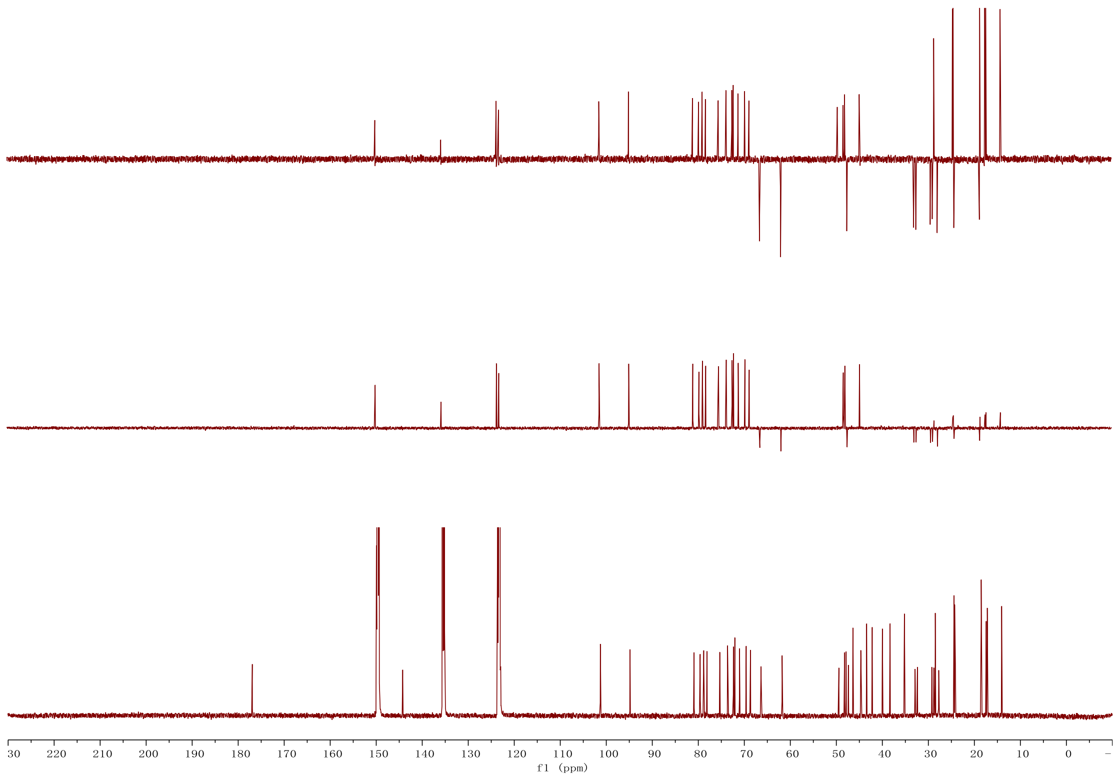

Figure S33 ^{13}C NMR spectrum of compound **6**(150 MHz, pyridine-d_5)

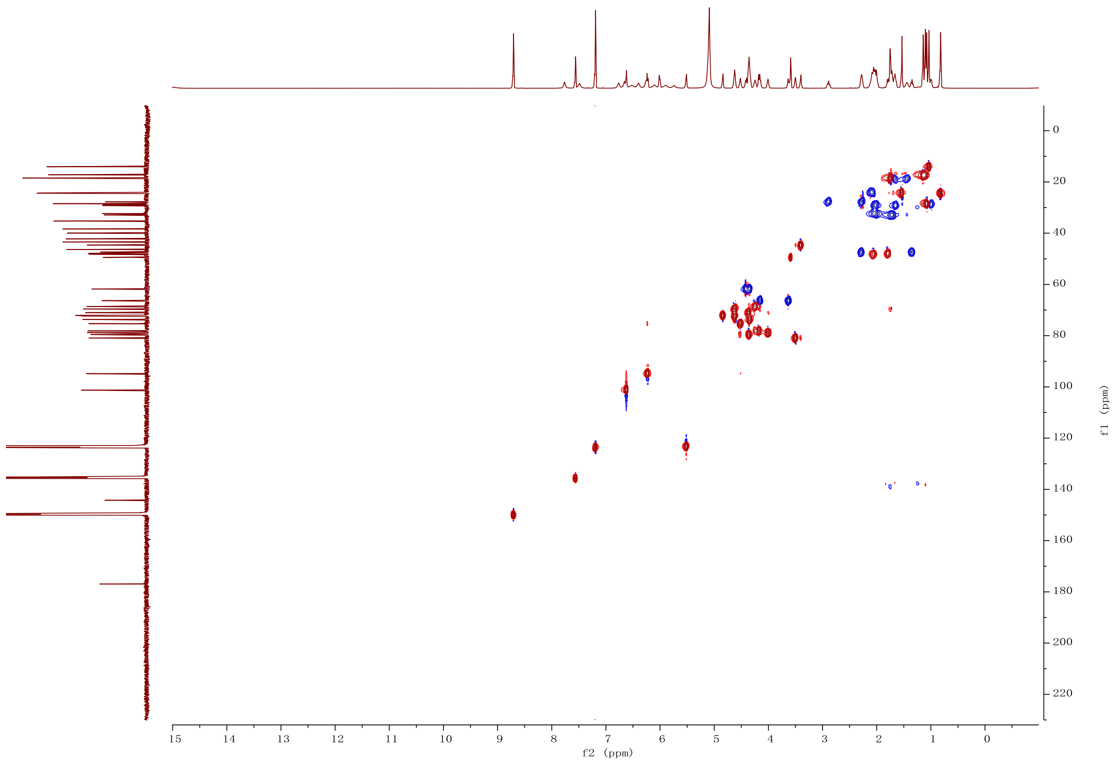

Figure S34 HSQC spectrum of compound **6**

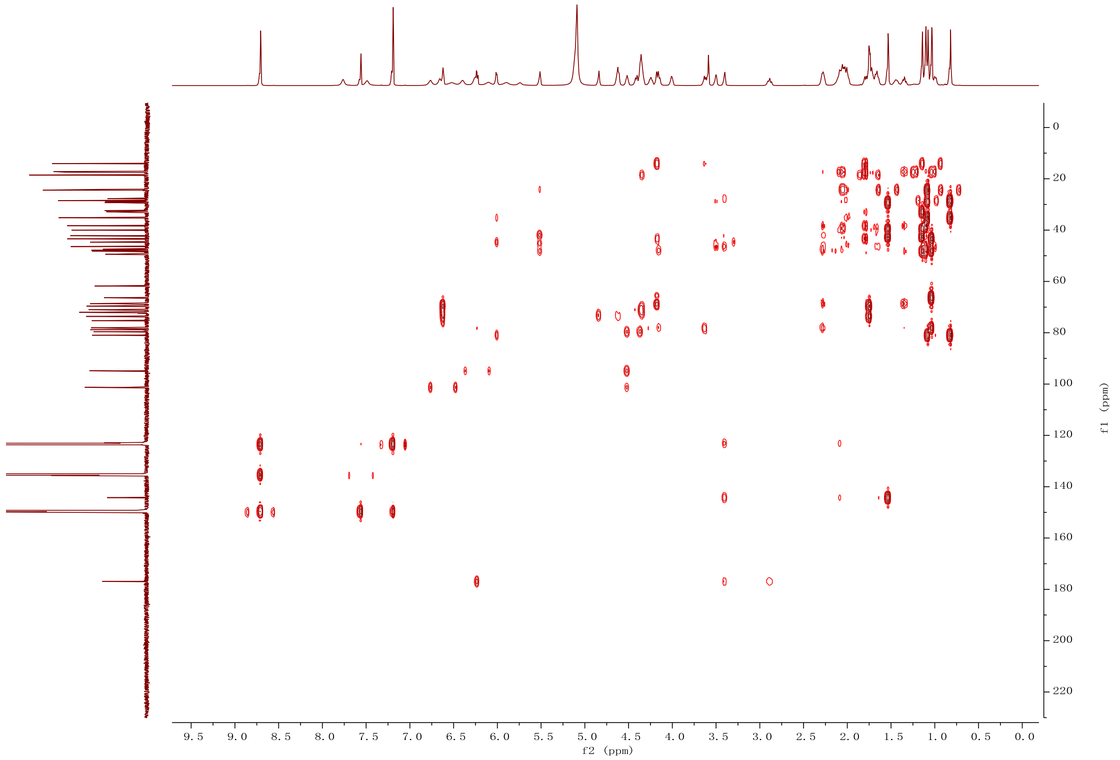

Figure S35 HMBC spectrum of compound **6**

Qualitative Analysis Report

**Data Filename**	fuya19.d	**Sample Name**	fuya19
**Sample Type**	Sample	**Position**	P1-C1
**Instrument Name**	Instrument 1	**User Name**	
**Acq Method**	s.m	**Acquired Time**	7/30/2019 11:49:39 AM
**IRM Calibration Status**	Success	**DA Method**	Default.m
**Comment**			

Sample Group **Info.**
Acquisition SW 6200 series TOF/6500 series
Version Q-TOF B.05.01 (B5125.2)

User Spectra

Fragmentor Voltage	Collision Energy	Ionization Mode
135	0	ESI

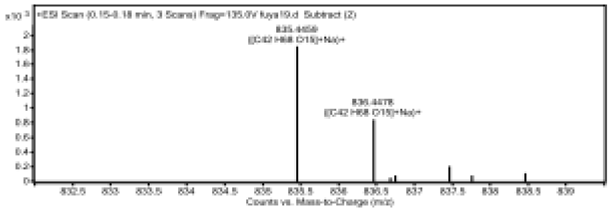

Peak List

m/z	z	Abund	Formula	Ion
102.1277	1	4945.02		
104.107		1894.2		
353.2642	1	1477.43		
369.2396		2653.37		
381.2961	1	1377.17		
397.2715	1	2374.74		
453.1668	1	1293.8		
575.3535	1	1042.65		
835.4459	1	1856.69	C42 H68 O15	(M+Na)+
836.4478	1	858.99	C42 H68 O15	(M+Na)+

Formula Calculator Element Limits

Element	Min	Max
C	3	120
H	0	240
O	0	30

Formula Calculator Results

Formula	CalculatedMass	CalculatedMz	Mz	Diff. (mDa)	Diff. (ppm)	DBE
C42 H68 O15	812.4558	835.4450	835.4459	-0.90	-1.08	9.0000

--- End Of Report ---

Figure S36 HRESIMS spectrum of compound **6**

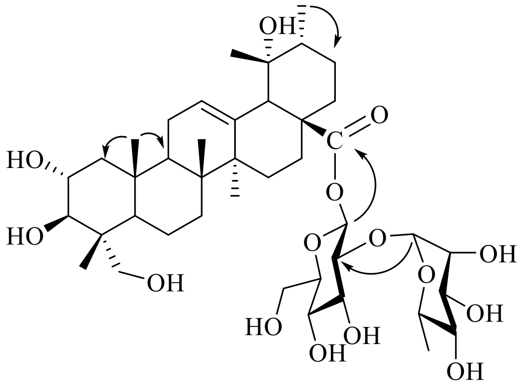

Figure S37 Key HMBC correlation of compound **7**

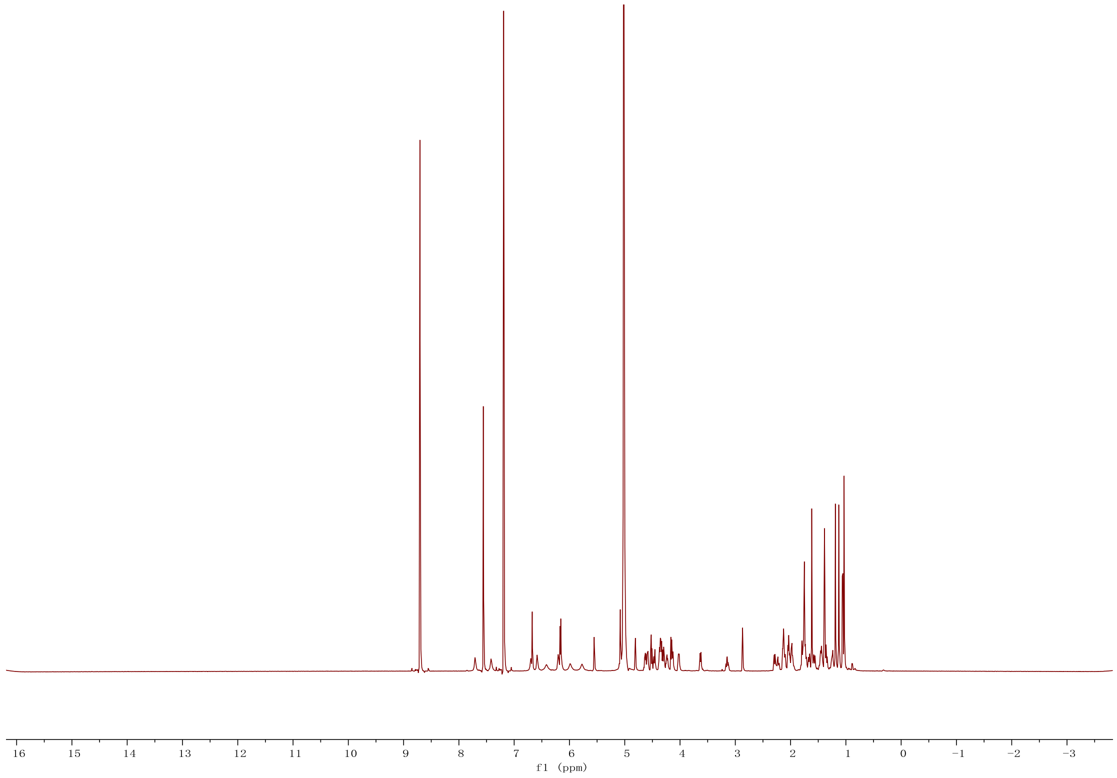

Figure S38 ^{1}H NMR spectrum of compound **7** (600 MHz, pyridine-d_5)

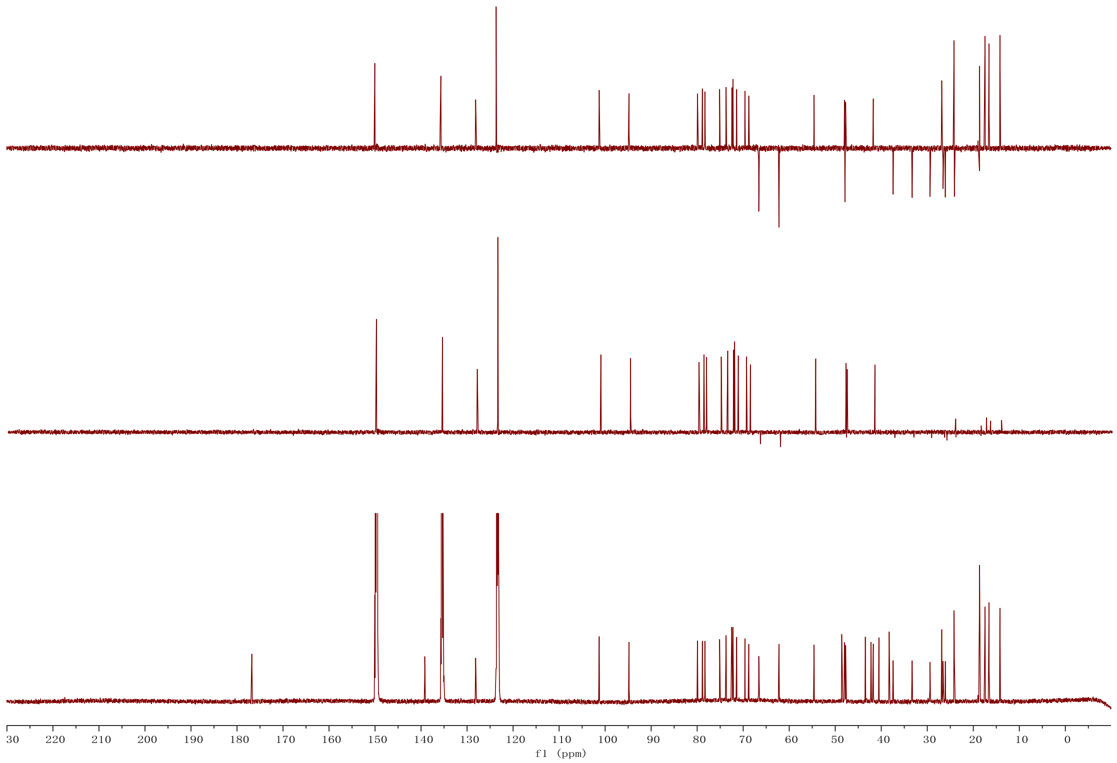

Figure S39 ^{13}C NMR spectrum of compound **7**(150 MHz, pyridine-*d*$_5$)

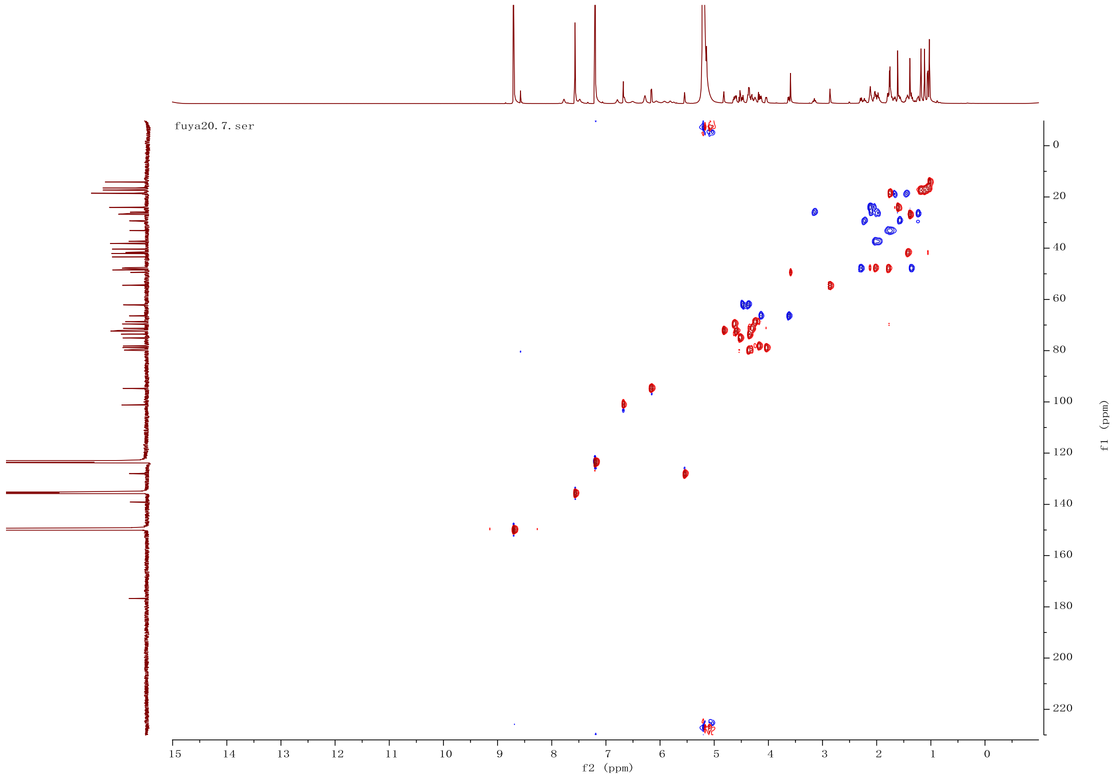

Figure S40 HSQC spectrum of compound **7**

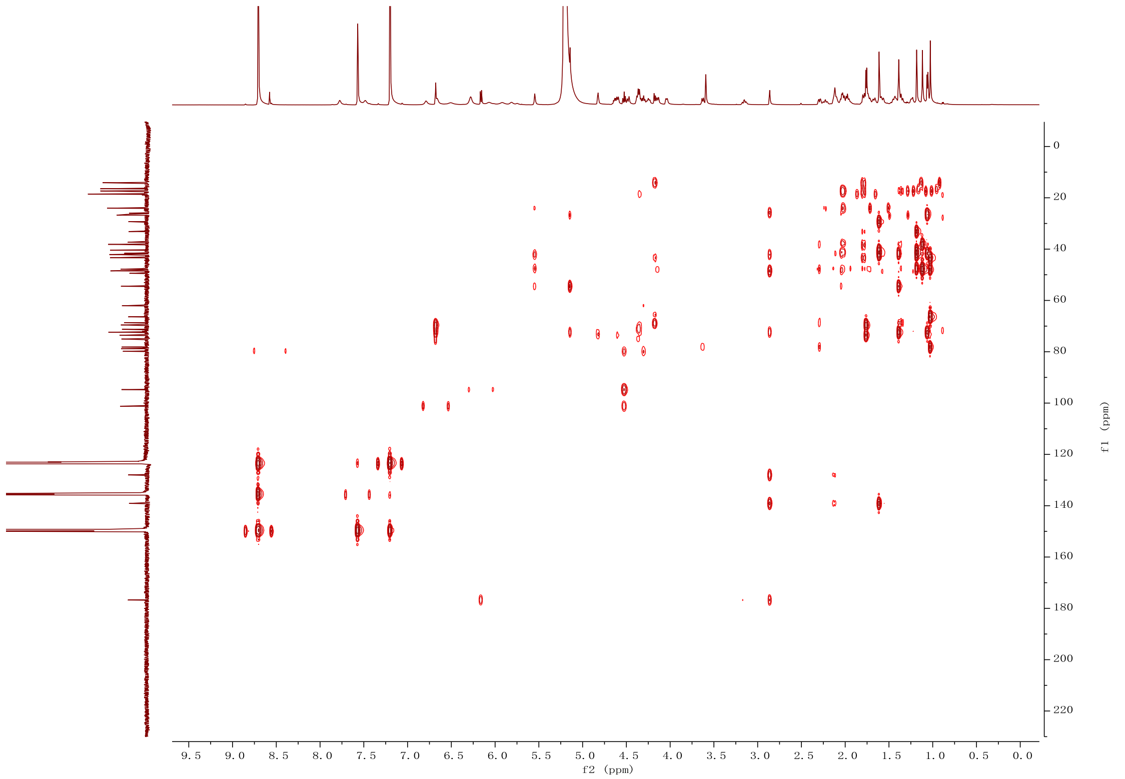

Figure S41 HMBC spectrum of compound **7**

Qualitative Analysis Report

**Data Filename**	fuya20.d	**Sample Name**	fuya20
**Sample Type**	Sample	**Position**	P1-C2
**Instrument Name**	Instrument 1	**User Name**	
**Acq Method**	s.m	**Acquired Time**	7/30/2019 11:50:49 AM
**IRM Calibration Status**	Success	**DA Method**	Default.m
**Comment**			

Sample Group **Info.**
Acquisition SW 6200 series TOF/6500 series
Version Q-TOF B.05.01 (B5125.2)

User Spectra

Fragmentor Voltage	Collision Energy	Ionization Mode
135	0	ESI

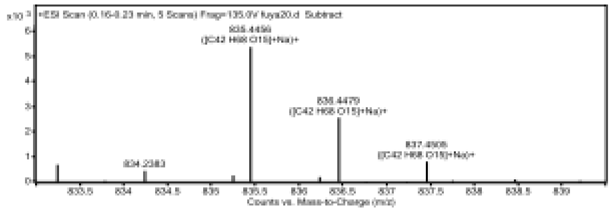

Peak List

m/z	z	Abund	Formula	Ion
102.1278	1	14985.8		
104.1071	1	12103.96		
118.1227		4621.56		
353.2661	1	4879.29		
355.2019	1	4754.04		
369.2402	1	6389.45		
381.2978	1	4499.81		
397.2712	1	5595.41		
453.1672	1	6818.02		
835.4456	1	5398.35	C42 H68 O15	(M+Na)+

Formula Calculator Element Limits

Element	Min	Max
C	3	120
H	0	240
O	0	30

Formula Calculator Results

Formula	CalculatedMass	CalculatedMz	Mz	Diff. (mDa)	Diff. (ppm)	DBE
C42 H68 O15	812.4558	835.4450	835.4456	-0.60	-0.72	9.0000

Figure S42 HRESIMS spectrum of compound **7**
